# The RNA-Binding Protein SMN as a Novel Player in Laryngeal Squamous Cell Carcinoma

**DOI:** 10.3390/ijms24021794

**Published:** 2023-01-16

**Authors:** Francesca Gabanella, Andrea Colizza, Maria Chiara Mottola, Silvia Francati, Giovanna Blaconà, Carla Petrella, Christian Barbato, Antonio Greco, Massimo Ralli, Marco Fiore, Nicoletta Corbi, Giampiero Ferraguti, Alessandro Corsi, Antonio Minni, Marco de Vincentiis, Claudio Passananti, Maria Grazia Di Certo

**Affiliations:** 1 CNR-Institute of Biochemistry and Cell Biology, Department of Sense Organs, Sapienza University of Rome, Viale del Policlinico 155, 00161 Rome, Italy; 2 Department of Sense Organs, Sapienza University of Rome, Viale del Policlinico 155, 00161 Rome, Italy; 3 Department of Experimental Medicine, Sapienza University of Rome, Viale del Policlinico 155, 00161 Rome, Italy; 4 CNR-Institute of Molecular Biology and Pathology, Department of Molecular Medicine, Sapienza University of Rome, Viale Regina Elena 291, 00161 Rome, Italy; 5 Department of Molecular Medicine, Sapienza University of Rome, Viale Regina Elena 324, 00161 Rome, Italy

**Keywords:** SMN, RNA-binding proteins, head and neck cancers, squamous cell carcinoma, EGFR

## Abstract

Head and neck squamous cell carcinoma (HNSCC) arises from the mucosal epithelium in the oral cavity, pharynx, sino-nasal region, and larynx. Laryngeal squamous cell carcinoma (LSCC) represents one-third of all head and neck cancers. Dysregulated RNA-related pathways define an important molecular signature in this aggressive carcinoma. The Survival Motor Neuron (SMN) protein regulates fundamental aspects of the RNA metabolism but, curiously, its role in cancer is virtually unknown. For the first time, here, we focus on the SMN in the cancer context. We conducted a pilot study in a total of 20 patients with LSCC where the SMN was found overexpressed at both the protein and transcript levels. By a cellular model of human laryngeal carcinoma, we demonstrated that the SMN impacts cancer-relevant behaviors and perturbs key players of cell migration, invasion, and adhesion. Furthermore, in LSCC we showed a physical interaction between the SMN and the epidermal growth factor receptor (EGFR), whose overexpression is an important feature in these tumors. This study proposes the SMN protein as a novel therapeutic target in LSSC and likely in the whole spectrum of HNSCC. Overall, we provide the first analysis of the SMN in human cancer.

## 1. Introduction

Head and neck squamous cell carcinomas (HNSCCs) represent a heterogeneous group of tumors that arise from the mucosal epithelium of the oral cavity, pharynx, nasal cavity and paranasal sinuses, and larynx [1]. They represent the sixth most common cancer worldwide [1,2]. In the oropharynx, SCCs are classified into human papillomavirus (HPV)-positive and HPV-negative subtypes, regarding their association with oncogenic strains of HPV. HPV-positive SCC has a more favorable prognosis than HPV-negative HNSCC [1]. Among the HPV-negative tumors, laryngeal squamous cell carcinoma (LSCC) represents 25% of all head and neck tumors and is the second most common malignancy after lung cancer [3]. Tobacco and alcohol abuse are known risk factors of HNSCC [4,5,6]. Carcinogenic-factor-mediated damage of the mucosal epithelium triggers genomic instability, the loss of tumor suppressor genes, and the activation of oncogenic signaling pathways, such as the epithelial growth factor receptor (EGFR) and phosphatidylinositol-3-kinase (PI3K)/AKT/mammalian target of rapamycin (mTOR). The overexpression of the EGFR is an important feature in HNSCC [7]. The EGFR is overexpressed in 80–90% of HNSCC tumors and is associated with poor overall survival and progression-free survival. Therefore, the molecular targeting of the EGFR by monoclonal antibodies, such as cetuximab, is a Food and Drug Administration (FDA)-approved therapeutic strategy for HNSCC patients [7]. However, patients with recurrent and metastatic disease rapidly develop resistance to cetuximab. Small molecules and oligonucleotides have also emerged as therapeutic inhibitors of key receptor-mediated signaling pathways, but such therapies have been disappointing in clinical trials as single agents. Despite advanced diagnostic tools, treatments, and clinical vigilance, the survival rate for HNSCC has not changed significantly in recent years [1,7]. Notably, laryngeal cancer is one of the few oncologic diseases in which the 5-year survival rate has decreased, even if minimally, from 66 to 63%, over the past 40 years [5]. This highlights the need for a further elucidation of the molecular signatures associated with this aggressive cancer type. Morphological and functional changes underlying tumor cell plasticity require multiple layers of gene expression control. In this context, a fine-tuning can occur through the action of RNA-binding proteins (RBPs), which promote a sophisticate gene expression control. Coherently, a dysregulation of RBPs has been linked to severe pathological conditions, including cancer [8]. For several RBPs (e.g., IGF2BP1, HuD, HuR, and nucleolin), a significant contribution to HNSCC has been established [9,10,11]. These proteins are physical/functional partners of the Survival Motor Neuron (SMN) protein. “RNA” is the keyword in SMN pathways [12]. The SMN protein was initially characterized once mutations in its coding gene, SMN1 (OMIM *600354), were linked to motor neurons’ degeneration in spinal muscular atrophy (SMA) [13,14]. It was subsequently established that SMN plays an essential role in all cell types [15]. By the formation and/or association with ribonucleoprotein (RNP) complexes, the SMN dictates important RNA processes, including the biogenesis of small nucleolar, nuclear, and Cajal body-associated RNPs; telomerase; and signal recognition particles. The SMN also acts in DNA repair, pre-mRNA splicing, transcription, mRNA trafficking, and translation [12]. Furthermore, recent studies highlighted a role of the SMN in the dynamic and composition of the cell surface [16,17]. It has been shown that the SMN interacts with caveolin-1, a structural component of the plasma membrane [16]. Interestingly, caveolin-1 has been identified as a biomarker to predict cetuximab response in patients with HNSCC [18]. Moreover, the SMN coexists with ribosomal proteins in caveolin-rich membrane domains and promotes local protein synthesis underlying the remodeling of the plasma membrane and cortical actin. This process requires a sophisticated interplay between the SMN and the mTOR pathway converging to local translation control [17,19]. The SMN is also known to drive peripheral traffic and the translation of β-actin mRNA and this event prevents the aberrant polymerization of the actin filaments [16]. Notably, a recent proteomics study about HNSCC biology highlighted not only an implication of RNA-related factors, but also an aberrant actin dynamic [20]. Based on these intriguing observations, we asked whether SMN itself could be implicated in biological and molecular aspects of HNSCC.

Here, we explored the expression levels of SMNs in laryngeal squamous cell carcinoma. We first performed a pilot study in a cohort of 20 patients with LSCC. We showed that the SMN is upregulated in LSCC tissues at both the transcript and protein levels. In HLaC-79 cells, a human LSCC cell line, the SMN impacts cancer-relevant behaviors, such as cell proliferation, cell migration, and cisplatin sensitivity. Furthermore, in agreement with our previous study [16], SMN-deficient HLaC-79 cells exhibit an aberrant actin dynamic. Moreover, we highlighted an intriguing link between the SMN and E-cadherin expression. Remarkably, in HLaC-79 cells, as well as in LSCC tissues, we found that the SMN physically contacts the EGFR. Collectively, this exploratory study points to the SMN as an attractive therapeutic target in HNSCC. Importantly, this is pioneering research regarding the role of the SMN in cancer.

## 2. Results

### 2.1. SMN Is Upregulated in LSSC

**Figure 1:**
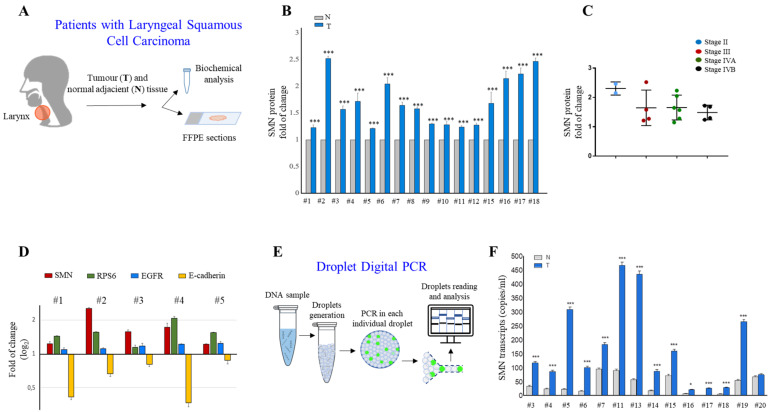
SMN is overexpressed in laryngeal squamous cell carcinoma. (**A**) Schematic representation of this pilot study. Within a cohort of 20 patients with LSCC, sample tissues from n = 16 patients were subjected to protein analysis, sample tissues from n = 14 patients were subjected to RNA analysis, and sample tissues from n = 2 patients to fluorescence microscopy. (**B**) Densitometric analysis of immunoblots using an anti-SMN monoclonal antibody. In each sample, SMN immunoreactivity was normalized to that of glyceraldehyde 3-phosphate dehydrogenase (GAPDH). SMN protein levels in the sample from tumor tissues (Ts) are expressed as the fold change compared with the sample from normal adjacent tissues (Ns). The number (#) identifies patients in Table 1. The graph illustrates the mean of three independent experiments. Error bars represent s.d. Asterisks indicate significative differences using unpaired *t*-test (*** *p* < 0.01). (**C**) Comparison of the SMN protein fold changes in different stages of laryngeal cancers. The box-and-whiskers graph shows the median, interquartile range, minimum, and maximum from the following number of patients per group: n = 2 for stage II; n = 4 for stage III; n = 6 for stage IVA; n = 4 for stage IVB. Data are analyzed by one-way ANOVA–Bonferroni’s multiple comparisons test. Mean ± s.d. are illustrated. No significative differences are observed between the different stages. (**D**) Densitometric analysis of immunoblots using antibodies against SMN, RPS6, EGFR, or E-cadherin. The immunoreactivity for each protein was normalized to that of GAPDH. Protein levels in tumor tissues (Ts) are ex-pressed as fold change compared with normal adjacent tissues (Ns). The mean of three independent experiments is shown in logarithmic scale (log2). Error bars represent s.d. (**E**) Diagram illustrating the main experimental steps of Droplet Digital PCR (ddPCR). (**F**) SMN mRNA expression levels (mean ± S.D.) measured by ddPCR comparing normal tissue (N) with tumor tissue (T) samples. The number (#) identifies patients in Table 1. Statistical significance of differences was evaluated by ANOVA followed by Bonferroni’s post-test: *** *p* < 0.001; * *p* < 0.05 (for each normal vs. tumor sample).

**Figure 2:**
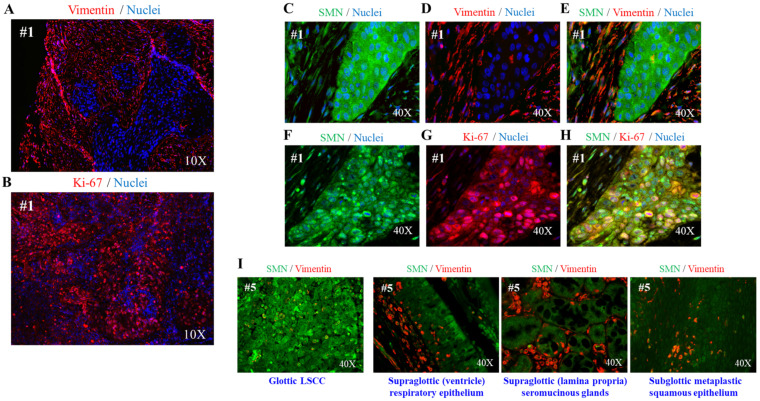
SMN protein is enriched in neoplastic cells of the laryngeal tissue. (**A**,**B**) Representative images of fluorescence microscopy. De-paraffinized sections of LSCC from patient #1 were stained with an anti-Ki67 antibody or an anti-vimentin antibody. Nuclei were labeled with DAPI (blue). Images were acquired with a 10× objective, as indicated. (**C**–**H**) Representative images of immunofluorescence analysis visualizing the localization of SMN protein. De-paraffinized sections of LSCC tissue from patient #1 were subjected to dual immunostaining for SMN (green) and vimentin (red) (upper panel, **C**–**E**), or SMN (green) and Ki67 (red) (bottom panel, **F**–**H**). Nuclei were stained with DAPI (blue). (**I**) Representative images of the immunofluorescence for SMN (green) and vimentin (red). Different types of epithelial cells identified in LSCC tissue from patient #5 are displayed. Images were acquired with a 40× objective.

**Figure 3:**
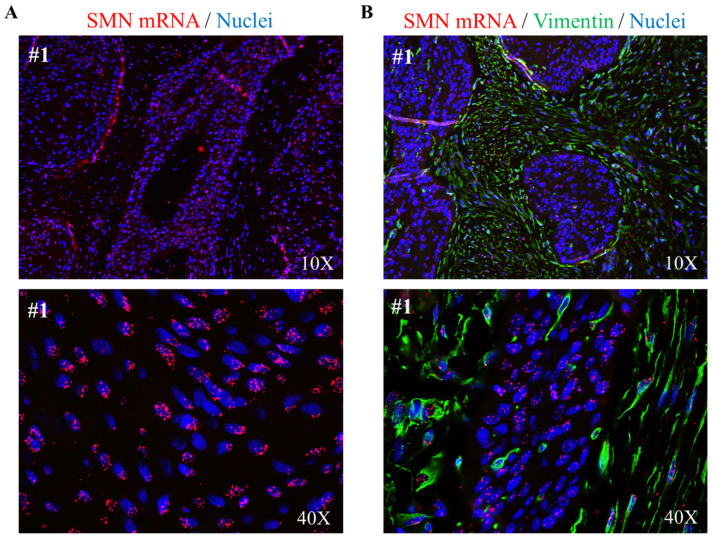
SMN mRNA localization in laryngeal squamous cell carcinoma. (**A**) Representative images of fluorescence microscopy. SMN mRNA (red dots) was detected by a padlock assay in LSCC tissue from patient #1. Nuclei were stained with DAPI (blue). Images were acquired with a 10× or 40× objective. (**B**) Representative images of fluorescence microscopy. LSCC sections were subjected to a combination of vimentin immunostaining (green) and a padlock assay targeting SMN mRNA (red). Nuclei were stained with DAPI (blue). Images were acquired with a 10× or 40× objective, as indicated.

Our intent was to explore a relationship between the RNA-binding protein SMN and LSCC. To this end, we conducted a pilot study in a cohort of 20 treatment-naive patients with LSCC (see Figure 1A). It is important to mention that LSCC represents one-third of all head and neck cancers [2]. For each patient, the tumor and normal adjacent tissue of the larynx were collected for biochemical assays. FFPE tissue samples from two patients (#1 and #5 in Table 1) were used for imaging studies. First, we evaluated SMN protein expression levels by Western blots and densitometric analysis. We tested and compared 16 LSCCs and their matched normal adjacent tissues (low-quality protein extracts were excluded from this analysis). As expected, the SMN was expressed in both normal and tumor laryngeal tissues. Remarkably, SMNs were significantly higher in all samples containing tumor tissue (Figure 1B). We asked whether SMN content was correlated with the clinical stage of LSCC. In particular, two patients (12.5%) were diagnosed with stage II, four patients (25%) were diagnosed with stage III, six patients (37.5%) with stage IVA, and four patients (25%) with stage IVB. Regarding this cohort of patients, we found no significant difference comparing SMN expression level changes among the tumor stages (Figure 1C). Although the number of patients was small, this result suggested that SMN dysregulation can impact on the biology of SCC, at least in the larynx. In a subgroup of patients (#1, #2, #3, 4#, #5), we also monitored other HNSCC-relevant proteins (Figure 1D and Figure S1). In addition to the EGFR, we tested the epithelial-to-mesenchymal transition (EMT) marker, E-cadherin, whose expression has been correlated with the clinicopathological features and patient outcome in LSCC [21,22]. In tumors, we detected an upregulation and downregulation of the EGFR and E-cadherin, respectively (Figure 1D and Figure S1). Overall, SMN changes were positively correlated with the EGFR regulation trend but negatively correlated with the E-cadherin regulation trend (Figure 1D). Furthermore, we were interested to monitor the ribosomal protein S6 (RPS6), a translational machinery component. We looked to RPS6 for at least two reasons: (1) it is known that an abnormal protein synthesis rate occurs in many cancer types [23,24]; (2) it has been demonstrated that the SMN controls translation machinery at multiple levels [16,17,19,25]. Notably, tumors displayed more abundant levels of RPS6 protein (Figure 1D and Figure S1). We speculated that dysregulated ribosome biogenesis could be a critical feature in LSCC. The next step was to quantify the SMN transcript by Droplet Digital PCR (ddPCR) (Figure 1E). This quantitative method provides an absolute amount of a target DNA in a biological sample, without the need for gene expression normalization [26]. As shown in Figure 1F, in comparison with normal adjacent tissues, the SMN transcript was significantly overexpressed in 13 tumors among a total of 14 tumors examined (low-quality RNA samples were excluded from this analysis). Therefore, in this cohort of LSCC patients, SMN was upregulated at both the transcript and protein levels. In addition to quantitative data, we approached imaging studies. FFPE sections of LSCC tissues were subjected to indirect immunofluorescence. Overall, in conventional SCC, vimentin immunostaining identifies stromal cells around and within the tumor nests. Conversely, tumor cells exhibit an immunoreactivity to the Ki-67 antibody. Ki-67 is a tumor marker that identifies active proliferation events and, most importantly, it correlates with the tumor aggressiveness in LSCC [27,28,29]. We confirmed the peculiar localization pattern of both vimentin and Ki-67, as shown in Figure 2A,B. To characterize the SMN localization pattern in LSCC, we subjected FFPE sections to dual immunostaining, combining the SMN antibody with vimentin or Ki-67 antibodies (Figure 2C–H). SMN staining generated a faint fluorescent signal in vimentin-positive cells (Figure 2C–E). Instead, SMN was strongly detectable in Ki-67-positive regions (Figure 2F–H). A comparison of the SMN staining in different regions identified within LSSC tissue (glottic LSCC, supraglottic respiratory epithelium, supraglottic seromucinous glands, subglottic metaplastic squamous epithelium), clearly suggested that SMN protein enriches in neoplastic cells (Figure 2I). We also detected a localization of EGFR and RPS6 proteins (Figure S2). In agreement with biochemical findings, fluorescence microscopy images confirmed the typical staining of the EGFR in this type of malignancy [30] and highlighted a potential dysregulation of translation machinery components. Furthermore, we also provide localization data of SMN mRNA. FFPE sections were subjected to a padlock assay. As previously reported, padlock assays allows the targeting of transcripts of interest in both fixed cells/tissues, with higher selectivity [19,31]. LSCC tissue samples were subjected to a padlock assay alone or in combination with vimentin immunostaining (Figure 3A,B). Fluorescence dots, corresponding to SMN mRNA, were visualized with higher frequency in tumor nests, where SMN transcripts appeared localized mainly at the perinuclear regions of the cells.

Collectively, by this pilot study we provide evidence that the SMN is overexpressed in LSCC.

### 2.2. SMN Knockdown Affects Cancer-Relevant Behaviors of HLaC-79 Cells

**Figure 4:**
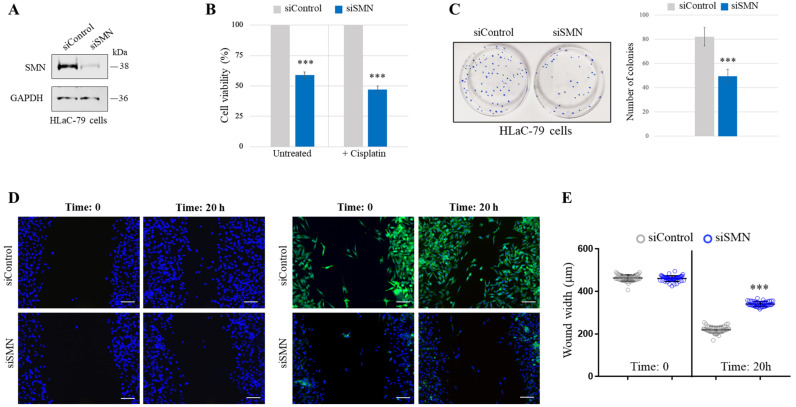
SMN knockdown affects cancer-relevant behaviors of HLaC-79 cells. (**A**) Western blot analysis of siControl- and siSMN-transfected HLaC-79 cells. Equal amounts of the protein extracts were checked for SMN and GAPDH. (**B**) siControl- or siSMN-transfected HLaC-79 cells were untreated or treated with 10 μg/mL cisplatin, for 24 h. Cell viability was determined by MTT assay. The percentage of viable SMN-deficient cells (siSMN) was calculated against the viability of the control cells, taken as 100% (siControl). The mean of three independent experiments is illustrated. Error bars represent s.d. Asterisks indicate significative differences using unpaired *t*-test (*** *p* < 0.01). (**C**) Colony formation assay comparing siSMN- and siControl-transfected HLaC-79 cells. Left, representative images of clones are shown. Right, the graph reports the number of clones per well. Data represent the mean of three independent experiments, performed in triplicate. Error bars represent s.d. Asterisks indicate significative differences using unpaired *t*-test (*** *p* < 0.01). (**D**) Representative images of wound healing experiments performed in siSMN-transfected HLaC-79 cells compared with the control (siControl). Cells were wounded by scratching the culture dish surface with a yellow pipette tip and then images were acquired at the times indicated. Nuclei were stained with DAPI (blue). The efficiency of SMN depletion was checked by immunofluorescence analysis using an anti-SMN antibody (green). Nuclei were labeled with DAPI (blue). Scale bar 100 μm (**E**) Multiple images of each wound were taken at the indicate experimental time. The wound width was measured along four different regions per field. In the graph are plotted all the results from three independent experiments. Mean ± S.D. are illustrated. Asterisks indicate significative differences using two-way ANOVA–Bonferroni’s multiple comparisons test (*** *p* < 0.0001).

To gain insights into the role of the SMN in LSCC, we conducted in vitro studies using HLaC-79 cells, a LSCC cell line [33,34]. For loss-of-function studies, we reduced the expression levels of endogenous SMNs by the transient transfection of HLaC-79 cells with SMN1-selective small interfering RNAs (siSMN). Scrambled siRNAs were used as a control (siControl) (Figure 4A). First, we evaluated the impact of SMNs on HLaC-79 survival and proliferation. We found that SMN knockdown affects the viability of HLaC-79 cells, as assessed by an MTT assay (Figure 4B, Untreated). Since cisplatin is the anticancer drug for advanced HNSCC, we also checked the viability of SMN-depleted cells upon cisplatin treatment (10 μg/mL) for 24 h. In our system, as revealed by an MTT assay (Figure 4B, +Cisplatin), SMN knockdown increased the sensitivity of HLaC-79 cells to cisplatin. In addition, we carried out a cell colony formation assay and observed that SMN knockdown reduced the colony-forming capability of HLaC-79 cells (Figure 4C). We next assessed the impact of SMN function on cellular migration. By a wound-healing assay, we quantified wound closure rates, 20 h after scratch, in both siSMN- and siControl-transfected cells. Remarkably, SMN knockdown significantly reduced the wound closure rate, in comparison with the control (Figure 4D,E), suggesting that the SMN contributes to the migratory capability of HLaC-79 cells.

### 2.3. SMN Impacts on the Regulatory Proteins of Cell Migration and Adhesion

**Figure 5:**
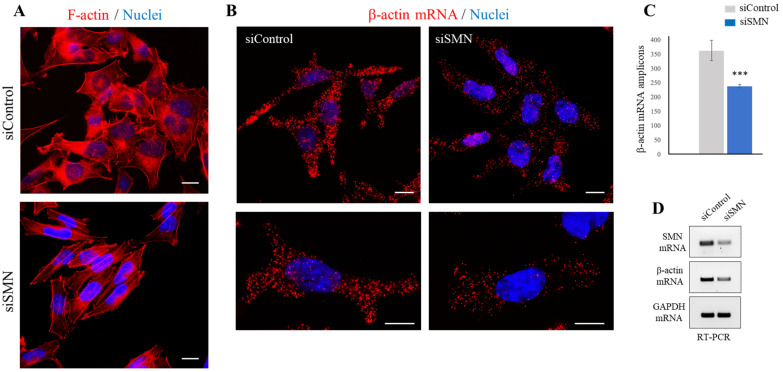
SMN knockdown dysregulates β-actin in HLaC-79 cells. (**A**) Representative images of fluorescence microscopy. siControl- and siSMN-transfected HLaC-79 cells were stained with Alexa Fluor 594-phalloidin to visualize the actin filaments (F-actin, red). Nuclei were labeled with DAPI. Scale bar 10 µm. (**B**) Padlock assay targeting β-actin mRNA (red dots) in siControl- and siSMN-transfected HLaC-79 cells. Nuclei were counterstained with DAPI (blue). Scale bar 10 µm. (**C**) Quantitative analysis of β-actin mRNA amplicons per cells (for each sample, a total of n = 15 cells were analyzed in each independent experiment). Data represent the mean of three independent experiments, performed in triplicate. Error bars represent s.d. Asterisks indicate significative differences using unpaired *t*-test (*** *p* < 0.01). (**D**) SMN mRNA, β-actin mRNA, and GAPDH mRNA in siControl- and siSMN-transfected HLaC-79 were checked by semiquantitative RT-PCR and analyzed by agarose gel electrophoresis. Panels are representative of three independent experiments.

It has been established that β-actin plays an essential role in regulating cell migration [35,36]. Notably, our previous study showed that SMN is required for the proper remodeling of actin filaments [16]. Based on these assumptions, we asked whether SMN knockdown may perturb actin dynamics in a laryngeal carcinoma cell line. To this end, we probed actin filaments (F-actin) with phalloidin and imaged cells by a fluorescence microscope (Figure 5A). Comparing siControl- and siSMN-transfected HLaC-79 cells, we observed that SMN knockdown changed actin cytoskeleton organization. As showed in Figure 5A, F-actin staining was reduced upon SMN depletion, indicating defective actin filament polymerization. Given the functional and physical link between the SMN protein and β-actin mRNA [12,16], we explored the effects of SMN depletion on β-actin transcript in HLac-79 cells. Images generated by padlock assays suggested that SMN knockdown caused a reduction in β-actin mRNA (Figure 5B,C). To further validate this result, we checked and compared the abundance of β-actin mRNA by a semiquantitative RT-PCR (Figure 5D and Figure S3). The biochemical approach agreed with our padlock images, suggesting that SMN-deficient HLaC-79 cells undergo perturbed β-actin dynamics.

**Figure 6:**
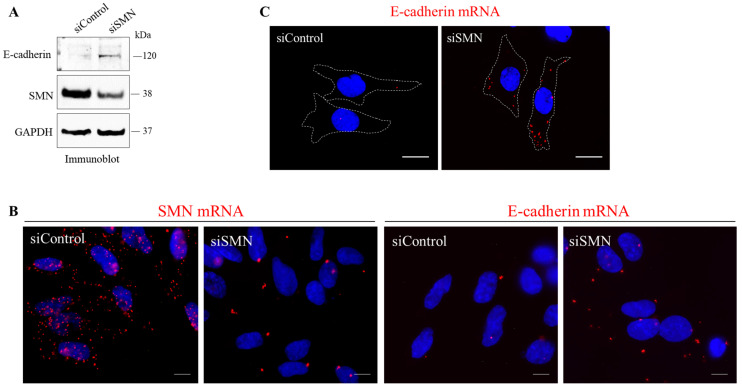
SMN depletion increases E-cadherin expression in HLaC-79 cells. (**A**) Western blot analysis of siControl- and siSMN-transfected HLaC-79 cells. Equal amounts of proteins were checked for E-cadherin and SMN. GAPDH was monitored as control of the protein loading. (**B**) Representative images of SMN mRNA (red dots) or E-cadherin mRNA (red dots) detected by padlock assay in siControl- and siSMN-transfected HLaC-79. Nuclei were labeled with DAPI. Scale bar 10 μm. (**C**) Representative images visualizing the E-cadherin mRNA (red dots) in siSMN- and siControl-transfected HLaC-79 cells. Outlines of cell surface are marked by a white dotted line. Nuclei were stained with DAPI. Scale bar 10 μm.

It is known that reduced E-cadherin expression allows the conversion of static and polarized epithelial cells into dynamic and invasive cells [37]. Notably, as above reported, LSCC exhibited a downregulation of E-cadherin protein, compared to normal adjacent tissues (Figure 1D and Figure S1). Therefore, in HLaC-79 cells we considered it interesting to explore a possible link between the SMN and E-cadherin. First, by Western blot we checked E-cadherin protein abundance in both siControl- and siSMN-transfected cells. In control cells, E-cadherin was almost undetectable. Interestingly, protein extracts from SMN-depleted cells were immunoreactive to the E-cadherin antibody (Figure 6A). To corroborate this result, we looked at E-cadherin mRNA. We designed padlock probes selectively targeting E-cadherin transcript or SMN transcript. Following padlock experiments, both siControl- and siSMN-transfected cells were visualized by fluorescence microscopy. Consistent with the siRNA-mediated silencing of SMN, images generated from the SMN padlock showed a strong reduction in the fluorescent dots per cell, in comparison to siControl-transfected cells (Figure 6B). In parallel, we probed E-cadherin mRNA. We observed that SMN knockdown caused an upregulation of E-cadherin mRNA (Figure 6B,C). Interestingly, as above reported, an inverse correlation between SMN and E-cadherin expression levels was also observed in LSCC (Figure 1). Collectively, these results provide evidence that SMN unequivocally plays a role in cell migration and adhesion in LSCC cells.

### 2.4. SMN Interacts with EGFR in LSCC

**Figure 7:**
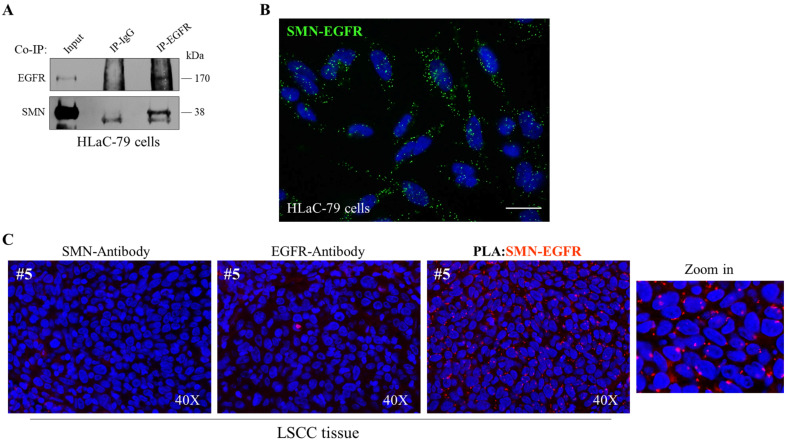
SMN interacts with EGFR in laryngeal squamous cell carcinoma. (**A**) Cellular extracts from HLaC-79 cells were processed for co-immunoprecipitation assay (Co-IP) using EGFR polyclonal antibody-conjugated beads (IP-EGFR) or rabbit IgG-conjugated beads (IgG), as negative control. Then, samples were subjected to Western blot analysis. The 5% of the protein extract was used as input. Representative immunoblotting of three independent experiments, showing the co-precipitation of SMN with EGFR. (**B**) Representative image of in situ proximity ligation assay (PLA) performed in HLaC-79 cells using primary antibodies against SMN and EGFR (mouse monoclonal antibody and rabbit polyclonal antibody, respectively). PLA puncta (green dots) are indicative of SMN-EGFR interaction sites. Nuclei were labeled with DAPI (blue). Scale bar 10 μm. (**C**) Representative image of in situ proximity ligation assay (PLA) performed in de-paraffined sections of LSCC tissue from patient #5, using primary antibodies against SMN and EGFR (mouse monoclonal antibody and rabbit polyclonal antibody, respectively). PLA puncta (red dots) are indicative of SMN-EGFR interaction sites. As negative control, PLA was performed using only one of the primary antibodies (anti-SMN or anti-EGFR). Nuclei were labeled with DAPI (blue). Images were acquired with a 40× objective.

EGFR overexpression occurs with high frequency in HNSCC [7]. Identifying modifier genes of the EGFR expression/pathway could improve therapeutic treatments for this aggressive cancer type. Given the ability of SMN to physically associate with cell surface proteins [16], we explored a potential SMN-EGFR interaction in the laryngeal carcinoma context. First, we subjected HLaC-79 cells to a canonical co-immunoprecipitation assay. As shown in Figure 7A, a pool of SMN protein co-precipitated with the EGFR, indicating a physical association between these proteins. The biochemical data were supported by co-localization images showing intracellular sites in which the EGFR and SMN signals appeared overlapped (Figure S4). We also performed an in situ proximity ligation assay (in situ PLA), which generates fluorescent dots in fixed cells only when two proteins are closer than 40 nm. Several PLA puncta were diffusely distributed within HLaC-79 cells (Figure 7B), indicating the existence of SMN-EGFR complexes. PLA images not only confirmed co-immunoprecipitation results, but also showed the ability of SMN protein to contact the EGFR in different cellular districts. Indeed, it is known that SMN shares different subcellular compartments with the EGFR, including the plasma membrane, cytoplasm, and nucleus [38]. Remarkably, an interaction between the SMN and EGFR in LSCC tissues was suggested by co-localization images (Figure S5) and confirmed by in situ PLA (Figure 7C). Even though preliminary and limited to LSCC, these findings identified the EGFR as a novel interaction partner of SMN.

Altogether, this pioneering study suggests that the RNA-binding protein SMN may be an intriguing player in LSCC and likely in the whole spectrum of head and neck tumors.

## 3. Discussion

RNA-binding proteins (RBPs) help to maintain cell homeostasis, tuning regulatory networks implicated in processes such as proliferation, differentiation, and metabolism [39]. Based on their biological functions, it is not surprising that alterations of RBPs occur frequently in cancer cells.

SMN influences the RNA life cycle at multiple levels, and it enables cells to finely regulate gene expression in time and space [12]. Due to its functional peculiarities, molecular strategies targeting the SMN in cells or tissues may contribute to disrupt multiple pathways at once. A deficiency of SMNs causes SMA, a genetic disorder characterized by the degeneration of alpha motor neurons and progressive muscle weakness [14]. To date, the role of the SMN in cancer is virtually unknown.

RNA-related pathways seem to act as driving forces for HNSCC development [40]. Basic and clinical research identified cellular pathways underlying HNSCC biology. However, a deep elucidation of the molecular landscape of this cancer type is needed to provide new opportunities for therapeutic intervention.

Here, we provide for the first time a demonstration that SMN is overexpressed in LSCC, which represents one-fourth of all head and neck cancers [3]. By a pilot study conducted within a cohort of 20 LSCC patients, we showed that SMN is upregulated in tumor tissue, at both the transcript and protein levels. Concerning this cohort of patients, the SMN appears upregulated regardless of the tumor stage. This suggests that the SMN could be implicated not only in cancer progression but also in cancer genesis. Consistent with this, increasing evidence points to a role of the SMN in stem cell self-renewal and pluripotency establishment [41,42]. Curiously, by our RNA sequencing data, we found that, among others, the CD44 gene was differentially expressed in SMN-deficient fibroblasts (our unpublished data). CD44 is a cell surface glycoprotein used to isolate cancer stem cells in HNSCC [43]. Further studies are needed to verify whether and how the SMN impacts tumor-initiating pathways in this aggressive carcinoma. Another intriguing aspect of this exploratory study is the positive correlation between the SMN and both EGFR and RPS6 expression patterns. Regarding the EGFR, it is well acknowledged that this receptor is overexpressed in approximately 80–90% of HNSCCs and correlates with the poor overall survival and progression-free survival of patients [1]. Instead, a relationship between RPS6 expression levels and LSCC has not yet been reported. RPS6 is a major structural component of translation machinery. We assume that an upregulation of RPS6 in LSCC cells could be consistent with a boost of protein synthesis required to shape tumor-related proteome. Not surprisingly, mounting evidence indicates that an enhancement of ribosome biogenesis gives competitive advantages to cancer cells [23]. Notably, the SMN has been found to regulate distinct aspects of ribosome biology, ranging from biogenesis to the local translation of ribosomal proteins [16,17,19,25,44]. Based on this notion, it is reasonable to suppose an implication of the SMN in sustaining the dynamic switches of protein synthesis underlying LSCC phenotype. In our opinion, the ability to control local translation machinery components is an attractive feature of the SMN, especially in a cellular context in which “specialized” networks rely on “specialized” protein synthesis production.

Furthermore, a potential role of the SMN in HNSCC was corroborated by in vitro studies using a cellular model of human LSCC. We demonstrated that SMN is required to sustain cancer-relevant behaviors, such as cell proliferation and migratory capability. We also observed that SMN knockdown increases cisplatin sensitivity. This is a critical issue in the HNSCC context. Cisplatin is still the anticancer drug for advanced HNSCC. However, HNSCCs exhibit different levels of cisplatin resistance. Cisplatin administration in resistant patients could generate almost no beneficial effect but could increase the chance of adverse side effects and tumor progression. At this stage, we can only speculate that molecular strategies targeting SMN in HNSCC could modulate the resistance to cisplatin-based chemotherapy. To gain insights into cellular activities concerning the actin cytoskeleton, cell–cell contact, and cell migration, we explored the effects of SMN knockdown on E-cadherin and β-actin proteins. By an evaluation of E-cadherin expression levels in SMN knockdown cells, we provided an important novelty in the context of both the SMN and HNSCC. Our findings suggest that the SMN could be involved in mechanisms regulating E-cadherin expression. Noteworthy, this result agrees with our Western blot analysis, revealing that SMN expression levels are inversely correlated with E-cadherin changes in LSCC cells. This strongly suggests an implication of the SMN in the regulatory networks underlying cell adhesion and extracellular matrix platforms. We think that an interplay between the SMN and the E-cadherin pathway may be crucial, since a loss of E-cadherin has been reported to trigger epithelial–mesenchymal transition in several cancers, including HNSCC [45,46]. Future studies will focus on this important issue. Regarding β-actin, we demonstrated that SMN depletion also perturbs actin dynamics in LSCC cells. Indeed, in knockdown cells, we observed not only a defective rearrangement of the β-actin filaments, but also a significant reduction in its transcript. These results are in part not surprising since an intimate connection between the SMN and actin dynamics is a well-established concept (16). Most importantly, our findings appear in line with an elegant work reporting that SMN deficiency causes R-loops’ accumulation at the transcription termination sites of the β-actin gene [47]. R-loops are evolutionarily conserved structures consisting of a DNA-RNA hybrid and a displaced single-stranded DNA, which form physiologically during transcription [48]. A disturbance of R-loops’ occupancy and clearance from the chromatin has been observed in patients with neurological diseases and cancer [49,50,51]. Zhao and co-workers showed that the SMN interacts with Senataxin, a major helicase, to resolve R-loops and targets it at the 3′ end of Polymerase II-transcribed genes [47]. This very important study highlighted a role of SMN in an R-loop resolution pathway. Given this notion, our ongoing studies aim to identify a potential link between the SMN and the R-loop-mediated epigenomic landscape in HNSCC. Furthermore, Huang and colleagues reported that FAT1 is among the most frequently mutated genes in HNSCC. Notably, a deficiency of FAT1 has been linked to aberrant actin remodeling at the cell periphery, as well as impaired cell adhesion and cell polarity [52]. Accordingly, proteomic data and pathway enrichment analysis revealed that FAT1 genetic aberrations converge on dysregulated actin dynamics, which may contribute to poor prognosis in patients with HNSCC [20]. In this regard, it is important to mention that FAT1 appeared differentially expressed in a transcriptomic profile of SMN-deficient fibroblasts (our unpublished data). Finally, in LSCC we also provide a demonstration of a physical interaction between the SMN and EGFR. This result not only confirms a general propensity of the SMN to cooperate with cell surface proteins [16], but also suggests that the SMN could mediate EGFR tuning. However, E-cadherin has been found to regulate the localization and activity of the EGFR [53,54]. Keeping in mind our findings showing an impact of the SMN on E-cadherin expression, it is plausible to suppose that the SMN might operate at the crosstalk between the EGFR and E-cadherin, thus affecting tissue morphogenesis and cancer progression.

Overall, to our knowledge this is the first study focusing on the RNA-binding protein SMN in cancer. Here, we provide evidence that the SMN could play a pivotal role in HNSCC biology. Although the underlying molecular mechanisms need further characterizations, the SMN emerges as a new attractive therapeutic target in LSSC and likely in the whole spectrum of HNSCC.

## 4. Materials and Methods

### 4.1. Antibodies and Reagents

The following antibodies were used: anti-SMN mouse monoclonal antibody (cat. no. 610647, BD Transduction Laboratories; work dilution for Western blotting, 1:10,000); anti-SMN rabbit polyclonal antibody (cat. no. sc-15320, Santa Cruz Biotechnology, Dallas, TX, USA; work dilution for immunofluorescence, 1:200); anti-SMN rabbit monoclonal antibody (cat. no. ab108424, Abcam, Cambrige, UK; work dilution for immunofluorescence, 1:200); anti-GAPDH mouse monoclonal antibody (cat. no. sc-47724, Santa Cruz Biotechnology; work dilution for western blotting, 1:500); EGFR rabbit polyclonal antibody (cat. no. sc-03-G, Santa Cruz Biotechnology; work dilution for immunofluorescence, 1:200); anti-Ki67 mouse monoclonal antibody (cat. no. MAB190, Millipore, Temecula, CA, USA; work dilution for immunofluorescence, 1:100); anti-RPS6 mouse monoclonal antibody (cat. no. sc-74459, Santa Cruz Biotechnology; work dilution for Western blotting, 1:1000; work dilution for immunofluorescence, 1:200); anti-E-cadherin mouse monoclonal antibody (cat. no. sc-71008, Santa Cruz Biotechnology; work dilution for Western blotting, 1:500); anti-vimentin mouse monoclonal antibody (cat. no. sc-6260, Santa Cruz Biotechnology; work dilution for immunofluorescence, 1:500); and anti-vimentin rabbit monoclonal antibody (cat. no. ab92547, Abcam; work dilution for immunofluorescence, 1:500). The secondary antibodies conjugated to horseradish peroxidase were purchased from Jackson ImmunoResearch Laboratories, Suffolk, UK, and used at a dilution of 1:5000. The Alexa Fluor488- and the Alexa Fluor594-conjugated secondary antibodies were purchased from Thermo Fisher Scientific Inc., Waltham, MA, USA, and were used at a dilution of 1:250. The Alexa-Fluor-594-conjugated phalloidin were from Life Technologies, Carlsbad, CA, USA. MTT, 3-(4,5-dimethylthiazol-2-yl)-2,5-diphenyltetrazolium bromide and cisplatin, cis-Diammineplatinum(II) dichloride were purchased from Sigma-Aldrich, Co., St. Louis, MO, USA.

The list of oligos used in this study is indicated in Supplementary Table S1.

### 4.2. Patients

A total of 20 patients with LSCC were included in this study. The sites of the tumors and staging and grading according to the American Joint Committee on Cancer [32] are summarized in Table 1. The study protocol conformed to the Declaration of Helsinki and its later amendments and was approved by the internal Institutional Review Board (Ethical Committee of Sapienza University and Policlinico Umberto I, Rome, Italy, approval number: 6462).

### 4.3. Cell Cultures and Transfections

A HLaC-79 laryngeal carcinoma cell line, kindly supplied from Marianne Schmidt, Klinik und Poliklinik für Hals-, Nasen- und Ohrenkranke Labor Josef-Schneider-Str. 11 97,080 Würzburg, was cultured in a RPMI 1640 medium (Gibco BRL, Grand Island, NY, USA) supplemented with heat-inactivated 10% FBS (Gibco), penicillin-streptomycin (Gibco), and GlutaMAX (Gibco), in a 5% CO_2_ humidified atmosphere, at 37 °C. For knockdown experiments, cells were transfected with 3 unique 27mer siRNA duplexes targeting the human SMN1 gene or with trilencer-27 scrambled negative control (OriGene Rockville, MD, USA). Lipofectamine 2000 (Life Technologies) was used as the transfection reagent, according to the manufacturer’s instructions. Cells were harvested after 48 or 72 h post transfection.

### 4.4. MTT Assay

Cell viability and proliferation were assessed by an MTT test. HLaC-79 cells (5 × 10^3^ cell/well) in a 96-well plate were transfected with siControl or siSMN siRNA and grown in a complete culture medium for 24 h. Next, cells were treated or not with 10 μg/mL cisplatin for a further 24 h. After incubation, MTT (3-(4,5-dimethylthiazol-2-yl)-2,5-diphenyltetrazolium bromide) was added to each well at a final concentration of 0.5 mg/mL. Following 3 h of incubation at 37 °C, dimethyl sulfoxide was added to dissolve the crystals. The absorbance was determined at 577 using a spectrophotometer microplate reader (NeoBiotech, Seoul, 08381, Republic of Korea).

### 4.5. Colony Formation Assay

For a colony formation assay, the HLaC-79 transfected cells were then harvested and seeded into a 60 mm plate at a density of 200 cells/well. The plates were cultured in a complete medium at 37 °C and 5% CO_2_ for 2 weeks, to allow colony formation. After washing in PBS, the colonies were fixed in 95% ethanol, air dried, and stained with Giemsa. Finally, the colonies were washed three time in water, dried counted, and imaged with a camera.

### 4.6. Wound Healing Assay

HLaC-79 transfected cells were cultured in 35 mm plates until a confluent monolayer was reached. A wound was created using sterile plastic disposable 200 µL pipette tips. Cells were washed twice to remove detached cells and cultured in a complete medium for 20 h. After fixing, cells were subjected to nuclear labelling with DAPI or immunofluorescence analysis with anti-SMN antibody. The healing distance was monitored with a conventional epifluorescence microscope (Olympus BX53; Milano, Italy), at 0 and 20 h. Images were captured by a SPOT RT3 camera and elaborated by IAS 2000 v.5.0.1 software (Biosistem ’82, Rome, Italy). The healing rate was quantified by measuring the distance between cells at the edges of the wound by using ImageJ, National Institutes of Health, USA 1.53a software.

### 4.7. Immunofluorescence

Formalin-fixed paraffin-embedded (FFPE) samples from two patients (#1and #5 in Table 1) were used for immunofluorescence studies. In one patient (#1, male, 63 years old) the SCC involved the glottis and was staged as IVA (T4aN0M0), according to AJCC [32]. In the other patient (#5, female, 75 years old), the SCC involved the supra-glottis and was staged as III (T3aN0M0), according to AJCC [32]. In both patients, the LSCC was graded as moderately differentiated. Immunofluorescence analysis was performed on four μm thick sections obtained from the FFPE blocks and loaded onto positively charged slides as previously described [31] with little modification. In brief: paraffin-embedded sections were dewaxed by 2 changes of xylene, 5 min each. After hydration in graded ethanol solutions (100%, 90%, and 70%, ethanol, for 2 min each), sections were incubated in the unmasking solution (10 mM sodium citrate, 0.05% Tween 20, pH 6.1) for 3 × 2 min and 4 × 30 s in a microwave oven at 750 Watt. After cooling to room temperature for 20 min, slides were rinsed in PBS and blocked with 1% BSA in PBS for 1 h at room temperature. Samples were incubated at 4°C overnight using the appropriate primary antibodies; washed three times in PBS/0.1% Tween 20; and incubated with the appropriate secondary antibodies. Slides were mounted with ProLong with DAPI (Thermo Fisher Scientific, Waltham, MA, USA) and examined by an epifluorescence microscope (Olympus BX53; Milan, Italy) equipped with a SPOT RT3 camera. Images were merged using the image analysis software IAS 2000 (Delta Sistemi, Alessandria, Italy).

Immunofluorescence analysis on fixed cells was performed as previously described [16].

### 4.8. Padlock Assay

Four μm thick sections obtained from the FFPE blocks were dewaxed in xylene, hydrated in graded ethanol solutions, and incubated in the unmasking solution as described above. After cooling to room temperature, slides were rinsed in PBS and incubated for 10 min on a magnetic stirrer with a solution of 0.5% acetic anhydride in 100 mM Tris-HCl (pH 8.0) to reduce nonspecific background. After two washes in PBS, slides were processed for the padlock assay as previously described [31]. When the padlock assay was combined with immunofluorescence analysis, slides were processed for the padlock assay, incubated with the appropriate primary antibodies, washed three times in PBS-0.1% Tween 20, then incubated with the appropriate secondary antibodies. In fixed cells, the padlock assay was performed as previously described [17].

### 4.9. In Situ Proximity Ligation Assay (PLA)

HLaC-79 cells and four μm thick sections obtained from the FFPE blocks were subjected to in situ PLA using Duolink In Situ Detection Reagents Green and Orange kit (DUO92014 and DUO920007, Sigma-Aldrich, Co., St. Louis, MO, USA.), according to the manufacturer’s instructions. A combination of primary antibodies to the SMN (mouse monoclonal antibody) and EGFR (rabbit polyclonal antibody) were used. The PLA signal was detected by a epi-fluorescence microscope (Olympus BX53; Milano, Italy).

### 4.10. Tissue Protein Extraction

A protein extraction was obtained from frozen tissues processed in a lysis buffer (1% SDS, 1% NP-40, 5% glycerol, 5 mM EDTA) supplemented with a complete protease and phosphatase inhibitor cocktail (cOmplete, EDTA-free Protease and PhosSTOP tablets, Roche, Indianapolis, IN, USA), using a homogenizer (7 mm, OMNI International GLH). The homogenates were boiled for 10 min and centrifugated for 20 min at 12,000× *g* at 4 °C. The protein concentration was measured by a QubitTM fluorometer (Invitrogen by Thermo Fisher Scientific Inc., Waltham, MA, USA), according to the manufacturer’s instructions. Protein extracts were stored at −80 °C until use.

### 4.11. Cellular Protein Extraction

HLaC-79 cells were processed in a lysis buffer (100 mM Tris-HCl pH 8.0, 150 mM NaCl, 5 mM EDTA, 1% Triton X-100) supplemented with a complete protease and phosphatase inhibitor cocktail (cOmplete, EDTA-free Protease and PhosSTOP tablets, Roche, Indianapolis, IN, USA). Extracts were passed five times through a 25 G needle, incubated on ice for 15 min, and clarified at 12,000× *g* for 10 min at 4 °C. The protein concentration was measured by a QubitTM fluorometer (Invitrogen), according to the manufacturer’s instructions. Protein extracts were stored at −80 °C until use.

### 4.12. Western Blot Analysis

Protein extracts were electrophoresed through standard 10% SDS-PAGE or NuPAGE 4–12% (Life Technologies Corporation) and transferred onto nitrocellulose membranes (GE Healthcare; Milano, Italy). The immunodetection of the reactive bands was revealed by chemiluminescence (ECL kit, GE Healthcare) and analyzed by an iBright 1500 (Thermo Fisher Scientific Inc.).

### 4.13. Co-Immunoprecipitation

Cellular extracts were prepared in an IP Buffer (50 mM Tris-HCl pH 7.5, 250 mM NaCl, 5 mM EDTA, 50 mM NaF, 0.1 mM NaVO4, 0.1% Triton X-100, 5% glycerol) and a complete protease and phosphatase inhibitor cocktail (cOmplete, EDTA-free Protease and PhosSTOP tablets, Roche, Indianapolis, IN, USA). Immunoprecipitation assays were performed overnight at 4 °C following the standard procedure, using the anti-EGFR rabbit polyclonal antibody. As a negative control, the immunoprecipitation was carried out with rabbit IgG beads (Thermo Fisher Scientific Inc.). After five washes in an IP buffer, the immunoprecipitated complexes were eluted by boiling in a Laemmly’s buffer for 10 min and analyzed by SDS-PAGE on 10% polyacrylamide gel followed by immunoblotting.

### 4.14. RNA Extraction, Retrotranscription, and Semiquantitative PCR

The total RNA from the cells and frozen tissues was extracted using TRIzol^®^ reagent according to the manufacturer’s instructions. The RNA from the HLaC-79 cells was then reverse transcribed using a High-Capacity cDNA Reverse Transcription kit (Thermo Fisher Scientific, Inc., Waltham, MA, USA). A semiquantitative PCR (RT-PCR) assay was performed in triplicate using the BioMix 2X (Bioline, Memphis, TN, USA) according to the manufacturer’s instructions. The RNA from the frozen tissues was treated to remove residual amounts of genomic DNA by DNase treatment, according to the following protocol: 4 μg of RNA were incubated with 1.4 units of DNase I (New England Biolabs, Ipswich, MA, USA) at 37 °C for 10 min. The sample was then treated with 1× EDTA (5 mM, pH = 8.0, Sigma-Aldrich, St. Louis, MI, USA) at 75 °C for 10 min, to deactivate the enzyme. Afterwards, 1 μg of DNase-treated RNA was reverse transcribed using the iScript cDNA Synthesis kit (Bio-Rad, Herules, CA, USA), containing both oligo-dT and random primers, according to the manufacturer’s protocol.

### 4.15. Droplet Digital PCR Expression Analysis

Droplet Digital PCR was performed using a 1× QX200 EvaGreen ddPCR Supermix (Bio-Rad), 100 nM of each primer, and 0.5 µL of each cDNA sample, according to the supplier’s specifications. Water-in-oil droplets were generated from the sample using the QX200 Droplet Generator (Bio-Rad) with its microfluidics system. The PCR was successively carried out using a C1000 Touch thermal cycler (Bio-Rad) following the Bio-Rad standard amplification protocol. Finally, the droplets underwent the QX200 Droplet Reader (Bio-Rad) and QuantaSoft software version 1.7.4 (Bio-Rad) was used to analyze the results.

### 4.16. Quantification and Statistical Analysis

All experiments were performed on at least three independent biological replicates. Data are presented as mean ± S.D. Statistical analysis was performed using the GraphPad Prism 9.4.1 software. Data were analyzed using an unpaired *t*-test or one-way or two-way ANOVA test with a Bonferroni test for multiple comparison as specified in the figure legends; *p* < 0.01 was considered statistically significant.

## Figures and Tables

**Table 1:** Clinical synopsis of the patients with LSCC included in this study. TNM, stage, and grade were based on AJCC [32]. A pack-year (PY) is used to define how many cigarettes you have smoked in your lifetime, with a pack equal to 20 cigarettes. Currently, having 20 pack-years or more is one of the criteria that needs to be met to be recommended for screening. Alcohol units per week (AUPW) define the quantity of pure alcohol in a drink. One unit equals 10 mL or 8 g of pure alcohol, which is around the amount of alcohol the average adult can process in an hour.

#	Gender	Age	Tumour Location	pTNM Stage	AJCC Stage	G	Exposure to Risk Factors: Alcohol	Exposure to Risk Factors: Tobacco
1	M	63	Glottis	pT4aN0M0	IVA	G2	NO	25 PACK-YEARS
2	M	59	Glottis	pT3N1M0	III	G2	8.4 AUPW	70 PACK-YEARS
3	M	71	Glottis	pT4aN0M0	IVA	G2	NO	27.5 PACK-YEARS
4	M	62	Supraglottis	pT4aN3bM0	IVB	G3	63 AUPW	60 PACK-YEARS
5	F	75	Supraglottis	pT3N0M0	III	G2	NO	40 PACK-YEARS
6	M	77	Supraglottis	pT4aN0M0	IVA	G2	NO	45PACK-YEARS
7	M	61	Supraglottis	pT4aN1M0	IVA	G3	29.4 AUPW	40 PACK-YEARS
8	M	78	Glottis	pT3N0M0	III	G2	NO	NO
9	M	78	Supraglottis	pT3N3bM0	IVB	G2	10.5 AUPW	45PACK-YEARS
10	M	68	Glottis	pT4aN2bM0	IVA	G2	29.4 AUPW	40 PACK-YEARS
11	M	58	Glottis	pT3N3bM0	IVB	G2	52.5 AUPW	100 PACK YEARS
12	F	77	Glottis	pT3N0M0	III	G2	NO	7.5 PACK-YEARS
13	M	63	Glottis	pT4aN2aM0	IVA	G2	29.4 AUPW	157.5 PACK YEARS
14	M	56	Supraglottis	pT4aN1M0	IVA	G2	86.8 AUPW	35 PACK YEARS
15	M	73	Supraglottis	pT3N3bM0	IVB	G2	NO	62.5 PACK-YEARS
16	F	62	Supraglottis	pT2N0M0	II	G2	NO	40 PACK-YEARS
17	M	69	Glottis	pT4aN0M0	IVA	G2	29.4 AUPW	62.5 PACK-YEARS
18	M	76	Glottis	pT2N0M0	II	G2	NO	60 PACK-YEARS
19	M	65	Subglottis	pT4aN2bM0	IVA	G3	1.4 AUPW	45PACK-YEARS
20	M	60	Glottis	pT4aN3bM0	IVB	G3	NO	70PACK-YEARS

## Data Availability

The authors confirm that the data supporting the findings of this study are available within the article.

## Supplementary Materials

The following supporting information can be downloaded at: https://www.mdpi.com/article/10.3390/ijms24021794/s1.
