# Supplementary material for: The RNA-Binding Protein SMN as a Novel Player in Laryngeal Squamous Cell Carcinoma
Source: Int J Mol Sci. 2023 Jan 16;24(2):1794. doi: 10.3390/ijms24021794 (PMC9864193; doi:10.3390/ijms24021794)
Supplement: Supplementary file 1 [file ijms-24-01794-s001.zip › ijms-2134885-supplementary.pdf]

**Supplementary Table S1.** Oligos used in the study

| **Primer name** | **Primer sequence (5'- 3')** |
|---|---|
| GAPDH F | CATGAGAAGTATGACAACAGCCT |
| GAPDH R | AGTCCTTCCACGATACCAAAGT |
| β-actin F | CATGTACGTTGCTATCCAGGC |
| β-actin R | CTCCTTAATGTCACGCACGAT |
| SMN F | GCAGCTTCCTTACAACAGTGG |
| SMN R | TGAAGCAATGGTAGCTGGGT |
| Padlock Probe SMN | TCATACTTGTCACATTTTTTTTCTCAATTCTGCTACTTTACTAC CTCAATTCTGCTACTGTACTACTTTTTTGAGAGTGCCTGGGCG |
| Padlock Probe β-actin | TGCGGTGGACGATGGTTTTTTTCTCAATTCTGCTACTTTACTAC CTCAATTCTGCTACTGTACTACTTTTTTTCCGCCTAGAAGCATT |
| Padlock Probe E-cadherin | TAGCTCTCGGCGTCATTTTTTTCTCAATTCTGCTACTTTACTAC CTCAATTCTGCTACTGTACTACTTTTTTGGCACCGTGAACGTG |
| RCA Primer | AGTACAGTAGCAGAATTGAG |
| AlexaFluor 595 labelled Probe | CTCAATTCTGCTACTTTACTAC |

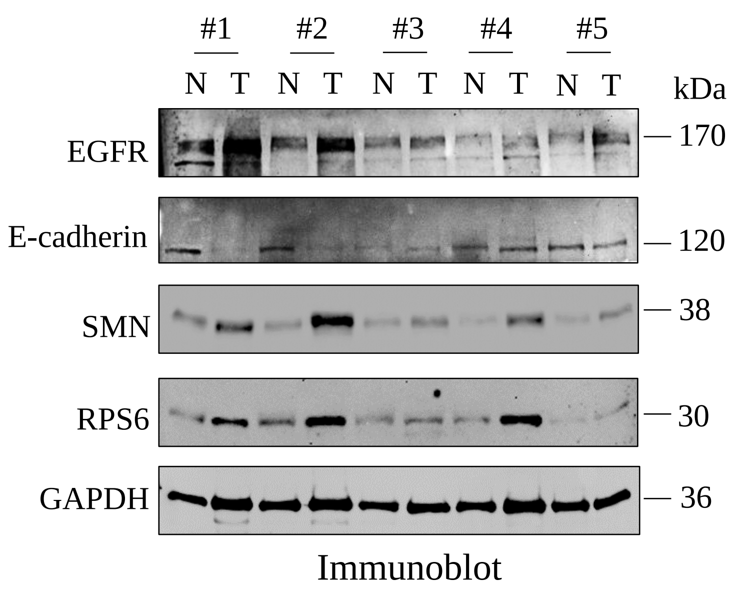


**Supplementary Figure S1. Analysis of SMN expression in laryngeal squamous cell carcinoma.** Representative western blot analysis of protein extracts from normal adjacent tissues (N) and tumour tissues (T) of patients with laryngeal squamous cell carcinoma (LSCC). The number (#) identifies patients in Table 1. Equal amounts of proteins were blotted and checked for EGFR, E-cadherin, SMN and RPS6. Levels of GAPDH were monitored as protein loading control.

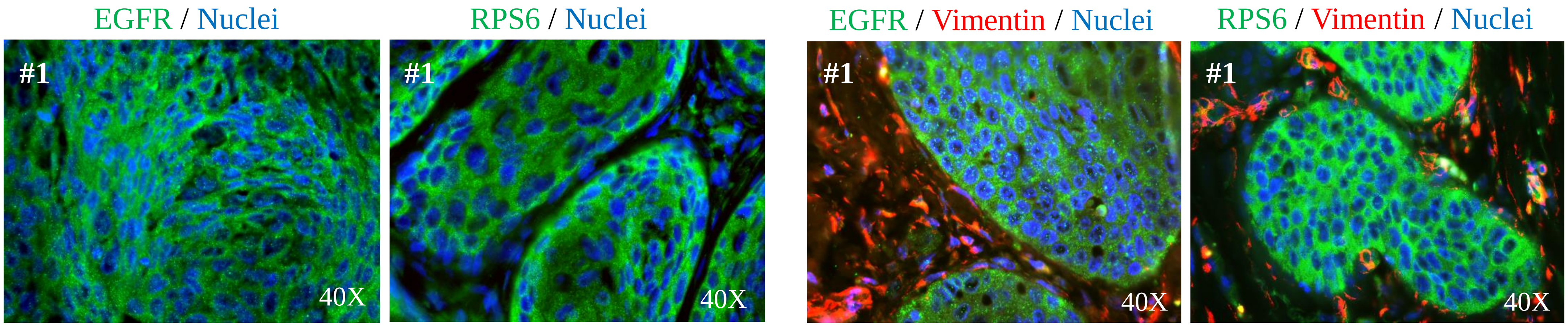


**Supplementary Figure S2. Representative fluorescence microscopy images of LSCC sections.** De-paraffined sections of LSCC tissue from patient #1 were immunostained with an anti-EGFR antibody (green), or an anti-RPS6 antibody (green), used alone or in combination with vimentin immunostaining (red), as indicated. Nuclei were stained with DAPI (blue). Images were acquired with a 40X objective.

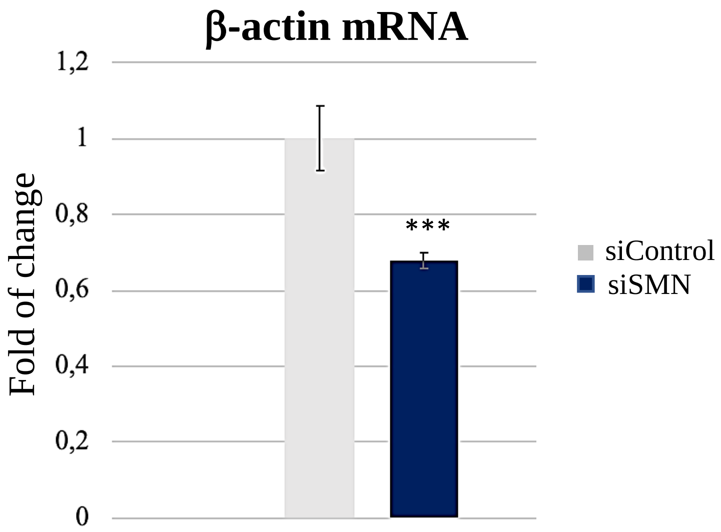


**Supplementary Figure S3. β-actin expression levels in SMN-deficient HLaC-79 cells.** Semiquantitative RT-PCR analyses evaluating β-actin mRNA normalized to GAPDH mRNA in siSMN- and siControl-transfected HLaC-79 cells. The graph illustrates the mean of three independent experiments. Error bars represent s.d. Asterisks indicate significative differences using unpaired t-test (*** $p < 0.01$).

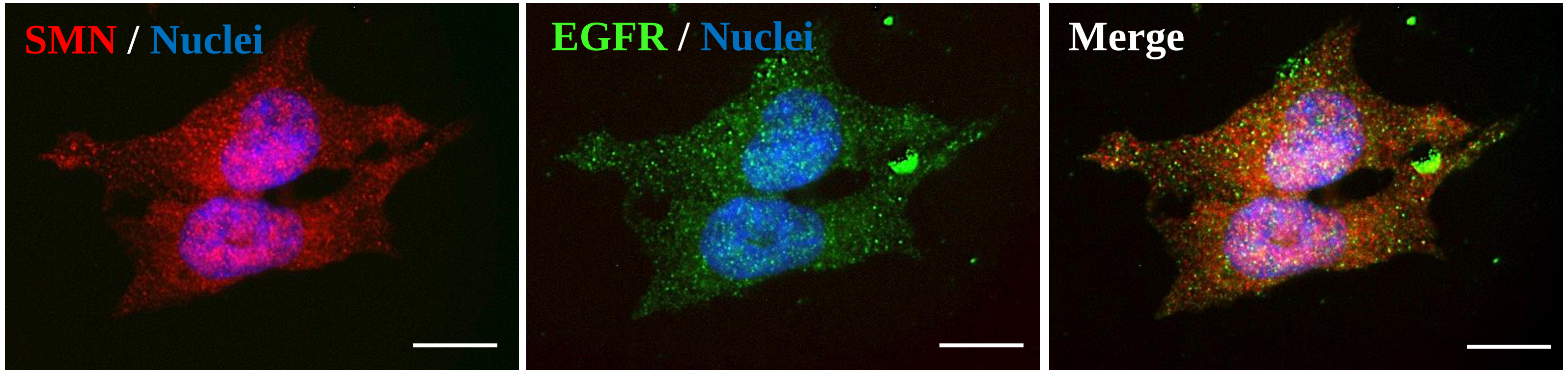


**Supplementary Figure S4. Representative fluorescence microscopy images.** HLaC-79 cells subjected to dual immunofluorescence for SMN (red) and EGFR (green). Nuclei were stained with DAPI. Scale bar 10 µm.

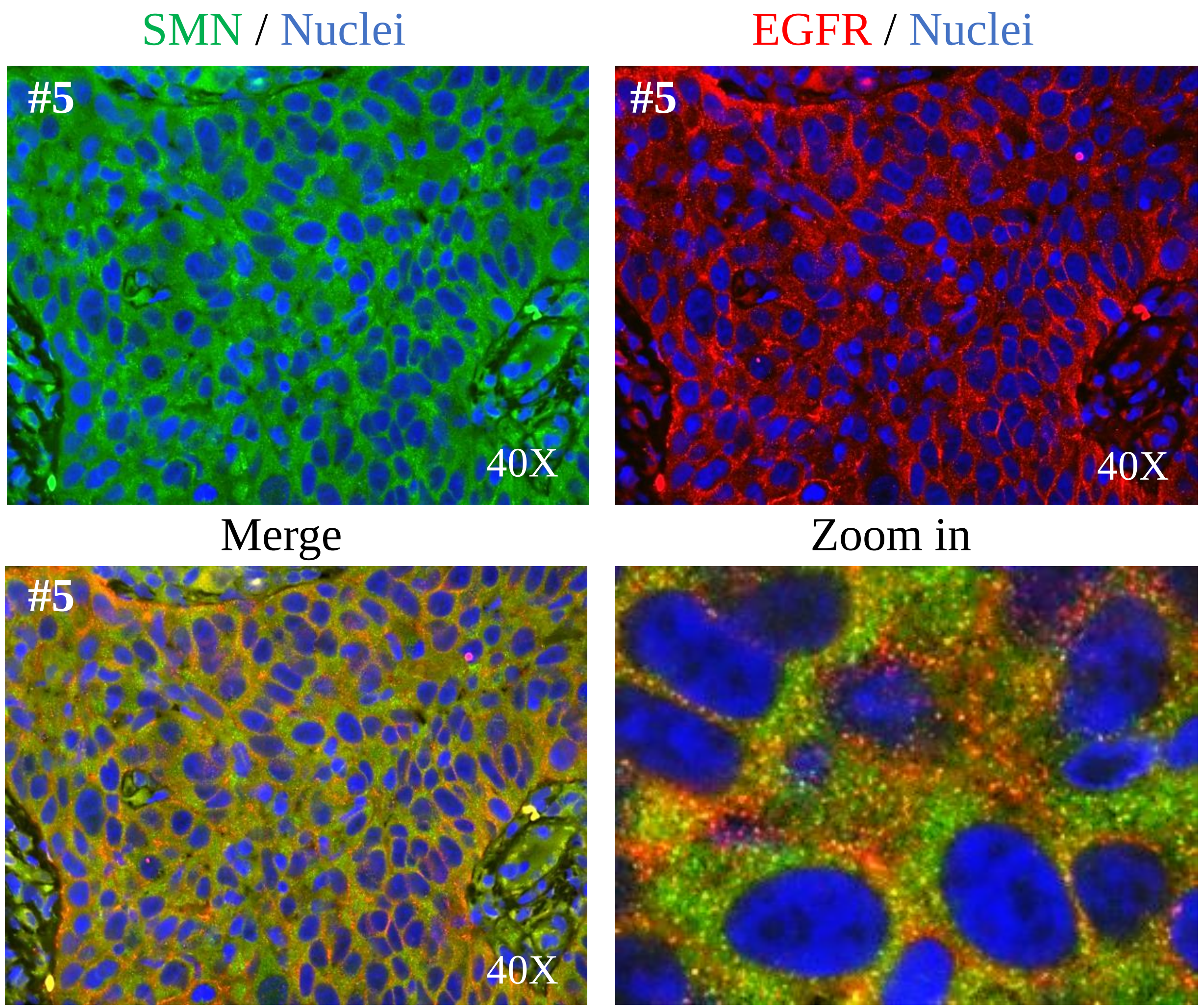


**Supplementary Figure S5. Representative images of SMN and EGFR localizations.** De-paraffined sections of LSCC tissue from patient #5 were subjected to dual immunofluorescence for SMN (green) and EGFR (red). Nuclei were stained with DAPI (blue). The images were acquired with a 40X objective.
